# Supplementary material for: Mesolimbic dopamine D2 receptors and neural representations of subjective value
Source: Sci Rep. 2019 Dec 27;9:20229. doi: 10.1038/s41598-019-56858-1 (PMC6934551; doi:10.1038/s41598-019-56858-1)
Supplement: Supplementary file 1 — Supplementary Information. [file 41598_2019_56858_MOESM1_ESM.pdf]

Supplementary Information

Title:

Mesolimbic dopamine D2 receptors and neural representations of subjective value

Authors:

Jaime J. Castrellon[1,2], Jacob S. Young[2], Linh C. Dang[3], Ronald L. Cowan[3,4,5], David H. Zald[3,4], Gregory R. Samanez-Larkin[1,2]

Affiliations:

[1]Department of Psychology and Neuroscience, Duke University; [2]Center for Cognitive Neuroscience, Duke University; [2]Department of Neurological Surgery, University of California, San Francisco; [3]Department of Psychology, Vanderbilt University; [4]Department of Psychiatry and Behavioral Sciences, Vanderbilt University School of Medicine; [5]Department of Radiology and Radiological Sciences, Vanderbilt University Medical Center

Supplementary Methods

fMRI modeling of individual differences in subjective value (SV) robustness-check

To evaluate the uniqueness of the association with subjective value signals, we conducted additional analyses that examined the robustness of the subject-specific effects in the present study. First, we tested whether mean SV-related BOLD signals reflect individual differences in discounting by estimating trial-to-trial SV from a group-estimated discount factor (k). Specifically, instead of fitting a hyperbolic discount function to each participant individually, we fit the model using all subjects' choice data as a single group, which resulted in a group parameter (k = .009679). We used this single k value to estimate trial-to-trial changes in SV of the chosen option for each participant and included this SV regressor as a parametric modulator of BOLD signal during the choice period (as in the primary analysis). Data was analyzed using the same mixed-effects general linear modeling in the primary analysis using FSL FEAT with group map thresholding using a cluster-forming threshold with a height of $Z > 2.3$, and cluster-corrected significance of $p < .05$. Across participants, mean activation was not associated with significant clusters in the frontal cortex or striatum. Peak effects emerged in the visual cortex and cerebellum (**Supplementary Figure S1**). Nevertheless, the correlation between D2 receptor (D2R) availability in the ventral striatum (VS) and SV-related BOLD parameter estimates in the ventromedial prefrontal cortex (vmPFC) was preserved (**Supplementary Figure S2**). Since rank-order participant differences in SV-related vmPFC parameter estimates were largely preserved across statistical maps based on either group-average or subject-specific discount rates, individual differences with D2R availability were also preserved

(**Supplementary Figure S2**). Prior studies of value-related neural activity using computational models have documented similar consistency of fMRI estimates across wide ranges of model-estimated parameters[1].

Next, to evaluate whether a value-model is required at all to observe individual difference associations between dopamine and fMRI BOLD activation, we ran a new mixed-effects general linear model that did not include a parametric regressor for SV and only evaluated mean activation during the choice period. Again, the group map was thresholded using a cluster-forming threshold with a height of $Z > 2.3$, and cluster-corrected significance of $p < .05$. Across participants, mean activation was not associated with localized clusters in the frontal cortex but instead activated a network of regions associated with task engagement in general (**Supplementary Figure S3**). Using this model, choice-related activation in the vmPFC was not correlated with D2R availability (**Supplementary Figure S4**). This provides additional evidence that subjective value modulation (estimated by an individual or group discount function) of the vmPFC and not mean activation is associated with dopamine function.

Supplementary Figure S1.

Mean effect of subjective value (N = 21) from a group-estimated discount factor overlaid on the mean participant T1-weighted image in standard space, whole brain cluster-forming threshold $Z > 2.3$, cluster-corrected $p < .05$.

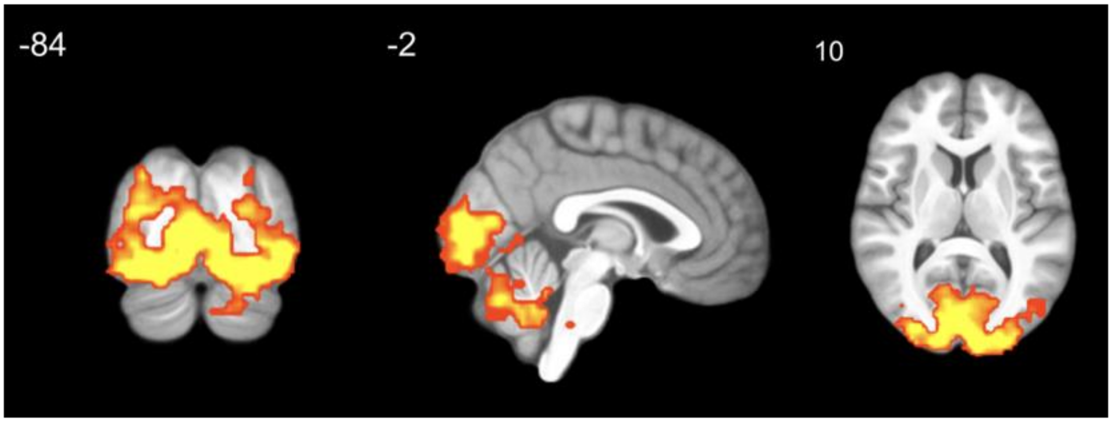

Supplementary Figure S2.

SV-related parameter estimates in the vmPFC were largely rank-order preserved when using a group-estimated discount rate or individual-estimated discount rate as evidenced by **A.)** the correlation between the shown on the left. As a result, **B.)** the correlation between VS D2R availability and SV-related parameter estimates in the vmPFC was preserved using a group-estimated discount rate.

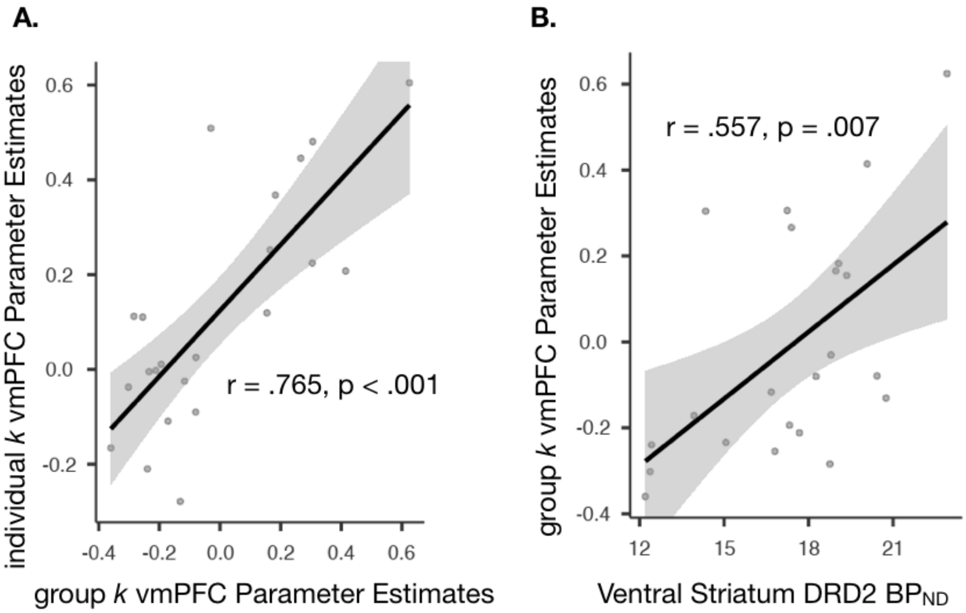

Supplementary Figure S3.

Mean activation during choice (N = 21) overlaid on the mean participant T1-weighted image in standard space, whole brain cluster-forming threshold Z > 2.3, cluster-corrected p < .05.

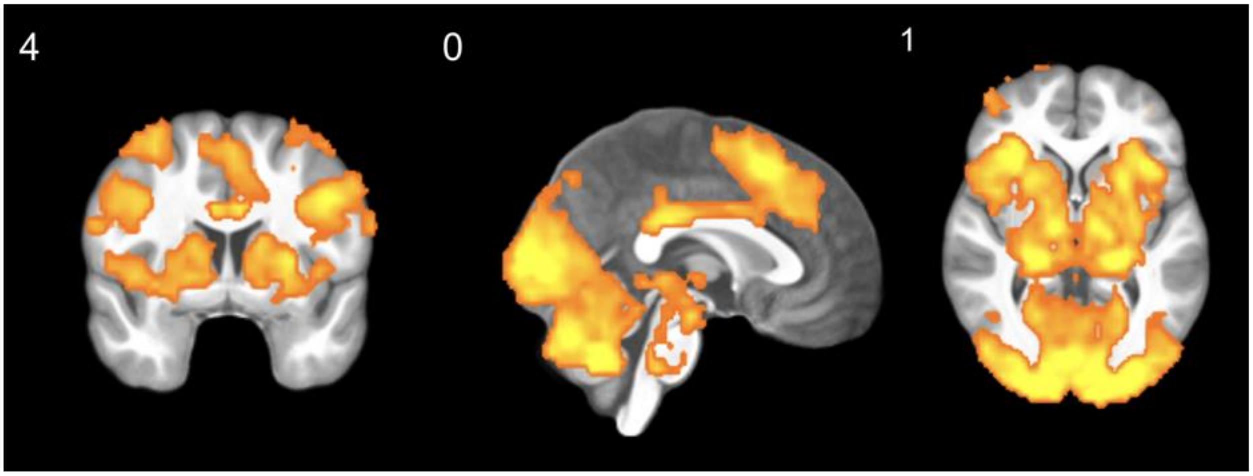

Supplementary Figure S4.

Mean BOLD activation in the vmPFC during the choice period was not correlated with VS D2R availability.

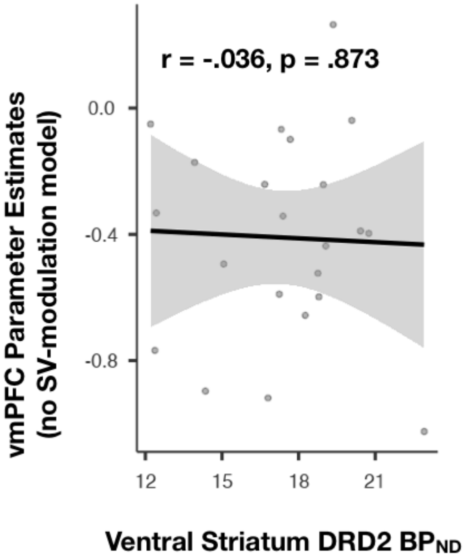

References

1 Wilson, R. C. & Niv, Y. Is Model Fitting Necessary for Model-Based fMRI? *PLOS Computational Biology* **11**, doi:10.1371/journal.pcbi.1004237 (2015).
